# Evidence for Selection in the Abundant Accessory Gene Content of a Prokaryote Pangenome

**DOI:** 10.1093/molbev/msab139

**Published:** 2021-05-08

**Authors:** Fiona J Whelan, Rebecca J Hall, James O McInerney

**Affiliations:** 1 School of Life Sciences, University of Nottingham, Nottingham, United Kingdom; 2 Institute of Microbiology and Infection, College of Medical and Dental Sciences, University of Birmingham, Birmingham, United Kingdom

**Keywords:** pangenome, microbial genomics, selection

## Abstract

A pangenome is the complete set of genes (core and accessory) present in a phylogenetic clade. We hypothesize that a pangenome’s accessory gene content is structured and maintained by selection. To test this hypothesis, we interrogated the genomes of 40 *Pseudomonas* species for statistically significant coincident (i.e., co-occurring/avoiding) gene patterns. We found that 86.7% of common accessory genes are involved in ≥1 coincident relationship. Further, genes that co-occur and/or avoid each other—but are not vertically inherited—are more likely to share functional categories, are more likely to be simultaneously transcribed, and are more likely to produce interacting proteins, than would be expected by chance. These results are not due to coincident genes being adjacent to one another on the chromosome. Together, these findings suggest that the accessory genome is structured into sets of genes that function together within a given strain. Given the similarity of the *Pseudomonas* pangenome with open pangenomes of other prokaryotic species, we speculate that these results are generalizable.

## Introduction

The mechanisms governing the existence of the pangenome—the totality of genes across a given set of genomes (Tettelin et al. 2005)—have been debated, with evidence being presented for both neutral and selective processes (McInerney et al. 2017a, 2017b; Shapiro 2017). Some evidence suggests that the accessory gene content within pangenomes has arisen as a consequence of extensive horizontal gene transfer (HGT) coupled with large effective population size and thus evolve neutrally (Andreani et al. 2017). In contrast, others argue that accessory genome evolution is dominated by selective pressures, and that diversity is maintained by selection acting on gene gain by horizontal acquisition, as well as gene loss (McInerney et al. 2017b; Bobay and Ochman 2018; Goyal 2018). Gene content changes therefore enable and are facilitated by population differentiation and niche adaptation (McInerney et al. 2017b; Goyal 2018).

Within, we argue that one way to determine the evolutionary pressures at play on the pangenome is to focus on gene–gene relationships within the accessory genome. If the pangenome is governed by neutrality, we would expect any observed structure in the accessory genome—including, for example, the co-occurrence of cofunctional genes—to have arisen by chance and thus to be rare. In contrast, if the accessory gene content is primarily or substantially shaped by natural selection, we would expect the accessory genome to be structured into groups of genes that work well together. Similarly, it would be reasonable to expect, at least in some cases, that genes whose interaction would be detrimental to the host to avoid being in the same genome. In this way, we expect that genes, which consistently co-occur or avoid each other across a large pangenome to be under selective pressures to maintain these patterns. As such, we use gene pair information to ask whether a portion of the accessory gene content is governed by selective pressure.

To answer this question, we use a previously published software called Coinfinder (Whelan et al. 2020) to focus on gene–gene association (i.e., co-occurrence) and dissociation (i.e., avoidance) patterns, collectively referred to as coincident relationships. We argue that, if evolving neutrally, we would not expect to see more coincident genes in the pangenome than would be expected by chance. In contrast, selective pressure would manifest as a significant proportion of the pangenome consisting of coincident gene relationships. In this case, we might further ask whether the assigned functionalities, gene expression patterns, and known protein–protein interaction partners of these genes also suggest co-selection. To conduct these analyses rigorously, we exclude genes that are vertically acquired. Coincident genes that are clade-specific (i.e., lineage-dependent genes) are likely to be coincident because they have remained within a single clade for the duration of their evolutionary history. Similarly, we exclude coincident gene pairs that share significant physical linkage (i.e., are colocalized on the genome) in order to ensure that the analysis is not focused on genes, which form functional units such as operons. Removing both of these types of genes provides us with a stringent set of lineage-independent coincident gene pairs with which to answer our research question.

In this paper, we focus on the genus *Pseudomonas*. Although pangenome analyses are typically conducted at the species-level, the *Pseudomonas* genus shares properties with other well-studied open pangenomes, including the ability to persist in a variety of niches (Stanier et al. 1966) and containing ample accessory gene content (approximately 81% in *Pseudomonas aeruginosa;*Kung et al. 2010; Ding *et al.* 2018). For example, although estimates vary based on data set size and analysis method, the accessory gene content (i.e., percentage of accessory vs. core genes across a genome set) of *Escherichia coli* is estimated to be between 86–91% (Ding et al. 2018; Decano and Downing 2019), *Streptococcus pneumoniae* 68–90% (Ding et al. 2018; Hiller and Sá-Leão 2018), and *Bacillus subtilis* 86% (Ding et al. 2018; Wu et al. 2021). In our analysis, we use coincident genes to ask whether the abundant accessory gene content of this microbial pangenome is maintained by selection. We identify coincident gene presence–absence patterns that deviate from random expectation and build network representations of the data to identify sets of coincident genes. We find that 86.7% of abundant accessory genes form ≥1 significant gene association/dissociation relationship. Co-occurring gene pairs are more likely to share functionality, be transcribed together, and to encode proteins that interact with each other more often than randomly paired accessory genes. Together, these results provide consilient lines of evidence supporting the hypothesis that selection on genome content drives the evolution of the abundant accessory pangenome of this prokaryote.

## Results

### Species and Gene Distribution in the *Pseudomonas sp.* Dataset

Two hundred and nine complete assemblies of *Pseudomonas* species were obtained from pseudomonas.com (accessed March 1 2019). The genomes were distributed across 40 *Pseudomonas* species, the most prevalent of which were *P. aeruginosa* (*n* = 81), *P. putida* (*n* = 18), *Ps. fluorescens* (*n* = 15), *P. syringe* (*n* = 13), and *P. stutzeri* (*n* = 10) (supplementary fig. 1*a*, Supplementary Material online). Twenty-five species were represented by a single genome within the data set. Furthermore, a total of 22 genomes were included that do not have a species identification.

Across these 40 species, we identified a total of 96,694 orthologous gene clusters (supplementary fig. 1*a*, Supplementary Material online). Of these, only 1,365 (1.41%) were identified in ≥90% of strains (i.e., “core” genes). The mean number of genes per genome was 5,530, meaning that in a given strain, an average of 24.9% of its genes are core. PAO1—a commonly studied *P. aeruginosa* lab strain (Klockgether et al. 2010)—was found to contain 5,601 genes (compared with 5,688 as annotated on pseudomonas.com), of which 1,494 are core genes. A total of 88,792 (91.8%) genes were found in ≤15% of genomes (supplementary fig. 1*a*, Supplementary Material online). Although the number of accessory genes varies across strains, the number of core genes is remarkably stable (supplementary fig. 1*b*, Supplementary Material online).

### The *Pseudomonas* Pangenome Contains an Abundance of Coincident Gene Relationships

**Fig. 1.**
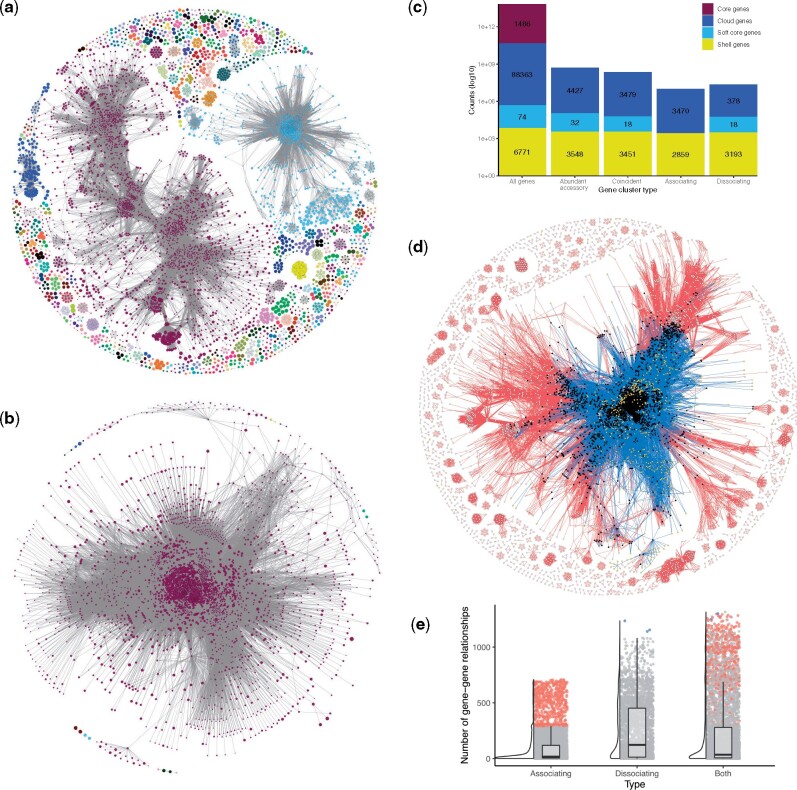
Network of coincident relationships in the *Pseudomonas* spp. accessory pangenome. Relationships between significantly associating (*a*) and dissociating (*b*) gene pairs are shown as gene–gene networks. Only nodes with a D≥−0.4 (i.e., sufficiently lineage-independent) are displayed. Nodes (i.e., gene clusters) are connected to other nodes if-and-only-if there is a significant coincident relationship between them. Nodes are colored by the connected component which they belong to; in other words, nodes are colored by significantly coincident gene sets. The size of the node is proportional to the D-value of the gene cluster (the larger the node, the more lineage-independent the gene is); the thickness of the edge is reversely proportional to the *P* value associated with the coincident relationship. (*c*) The counts of the genes present in the pangenome, the abundant accessory subset, as well as those which associate, dissociate, or form either type of relationship (i.e., are coincident). (*d*) A gene–gene network of all lineage-independent coincident gene relationships. Edges are colored by association (red) and dissociation (blue) relationships. Genes which form both association and dissociation relationships are represented by black nodes, genes which only associate by white, and genes which only dissociate by yellow. (*e*) The distribution of gene–gene relationships across genes. Boxplots display the first and third quartiles, with a horizontal line to indicate the median, and whiskers extend to 1.5 times the interquartile range. Associating and dissociating “hub” genes are colored.

Using the gene annotations provided by pseudomonas.com and gene clusters identified with Roary (Page et al. 2015), the 96,694 orthologous gene clusters (herein referred to as gene clusters) were used to identify coincident gene relationships within the pangenome. Any gene cluster that was considered core or present in ≤5% of strains were culled from coincident analyses, leaving 13,864 gene clusters across 209 genomes for testing. From these analyses (detailed in the Materials and Methods), we identified a *significantly associating dataset* comprised of 293,123 co-occurring gene pairs. We build a network representation of the gene pairs such that each gene is represented by a node, which is connected to another gene if those genes co-occur with each other. In this way, we identify 433 connected components or gene sets (fig. 1*a*). The 433 associating gene sets are well dispersed across the *Pseudomonas* spp*.* core gene phylogeny and none are species-specific, indicating the effect of culling lineage-dependent genes from the analysis (supplementary fig. 2, Supplementary Material online). Similarly, we determined the *significantly dissociative dataset* that contains 421,080 dissociative gene pairs organized into 13 connected components (fig. 1*b*).

Of the 13,864 accessory gene clusters identified in ≥5% of *Pseudomonas* strains (i.e., the abundant accessory genes tested by Coinfinder; Whelan et al. 2020), 8,007 (57.7%) were lineage-independent (see Materials and Methods, supplementary fig. 3, Supplementary Material online). Of these 8,007 clusters, 6,329 and 3,589 formed associating and dissociating relationships, respectively (fig. 1*c* and supplementary fig. 4, Supplementary Material online). Accounting for the genes involved in both types of relationships, a surprising 6,948 (86.7%) of abundant lineage-independent accessory genes were involved in ≥1 coincident relationship. Although gene dissociations were identified across all three noncore gene categories, gene associations were only identified in the two more rare gene categories (Cloud and Shell genes; fig. 1*c*). Similar results were found when both lineage-independent and -dependent genes were considered (supplementary fig. 5*a*, Supplementary Material online).

Of the 6,329 genes forming coincident relationships identified, 2,970 (46.9%) are involved in both association and dissociation relationships, meaning that they both co-occur with, and avoid other genes in the pangenome (fig. 1*d*; black nodes). These 2,970 dual-relationship genes account for 268,647 (91.6%) of all gene–gene associations and 418,698 (99.4%) of all gene–gene dissociations (fig. 1*d*). That is to say that almost half of the coincident genes account for the majority of coincident gene relationships. On average, associating genes form relationships with 94 and a median of 18 other genes (fig. 1*e*). However, the distribution is uneven, with 24.3% of genes forming fewer than five connections to other genes (1,542 genes < the 25th percentile; fig. 1*e*). The 624 association hubs (i.e., genes with >1.5× the upper interquartile range) each have ≥290 gene associations and account for 50.8% of the total observed gene association patterns. In contrast, dissociations in the *Pseudomonas* pangenome are driven by a small number of dissociation hub genes (*n* = 3) that each form ≥1,110 gene dissociation relationships. Among the associating and dissociating hub genes are a diversity of functions including transcriptional regulators, transporter subunits, metabolic enzymes, and an abundance of hypothetical proteins. Interestingly, for those genes that were found to have both types of coincident relationships, no gene acts as both an associating and dissociating hub (fig. 1*e*). The number of hub genes increases when lineage-dependent genes are included in these analyses (supplementary fig. 5*b*, Supplementary Material online).

### Colocalization of Coincident Genes

**Fig. 2.**
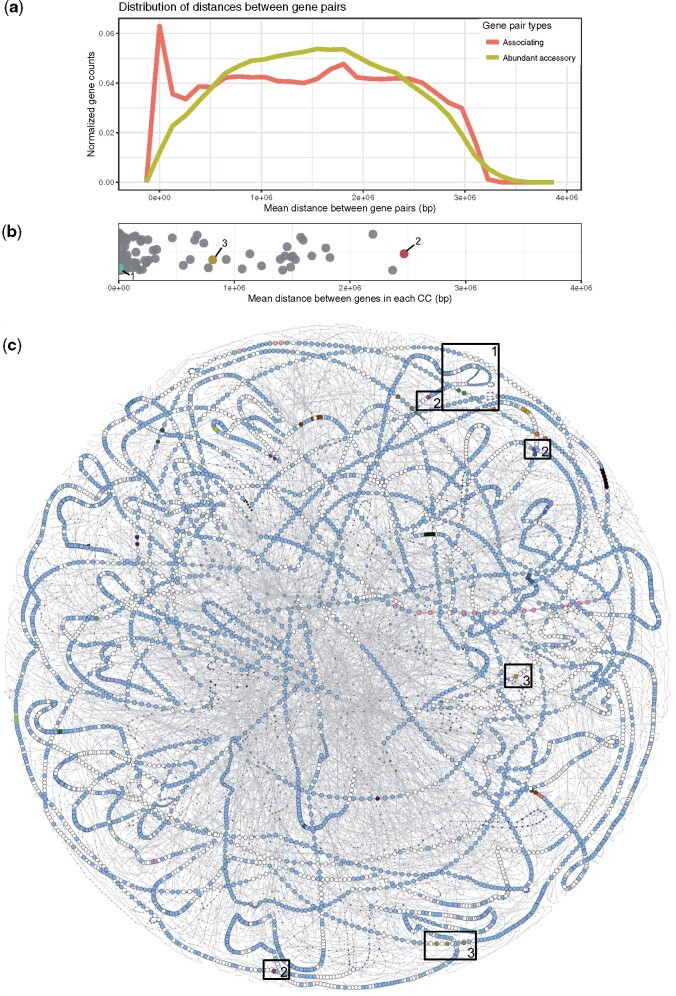
Colocalization amongst associating gene pairs. (*a*) Associating genes are more likely to be colocalized than are randomly assigned abundant accessory gene pairs on *Pseudomonas* spp. chromosomes. (*b*) Twenty-six percent of all sets of associating genes (i.e., connected components of genes which share co-occurrence patterns) do not share significant physical linkage as defined by the mean syntenic distance between all genes within a gene set. Colored gene sets correspond to labeled boxes in part C. (*c*) The pangenome graph of the *P. aeruginosa* subset of the *Pseudomonas* dataset. The pangenome graph of the full data set is available in supplementary figure 5, Supplementary Material online. Labeled boxes show examples of gene association clusters that are colocalized (box 1, turquoise genes), are not colocalized (boxes 2, red genes), and have variable levels of syntenic distance (boxes 3, green genes). For visibility, cloud genes are not shown.

HGT and differential gene loss are the main contributing factors to pangenome formation (Azarian et al. 2020). If functionally related gene pairs are found in close proximity on a genome, then they may have been acquired in a single HGT event, and their co-occurrence pattern might be a consequence of the HGT process, and not a consequence of natural selection. However, many known protein interactions occur between genes that are dispersed across the genome (e.g., proteins produced by genes *crr* and *ptsG* form the EII complex in enteric bacteria and are not in close proximity on the genome; Deutscher et al. 2006). To explore whether colocalization and the simultaneous transfer of genes are responsible for gene association relationships in the pseudomonads, we compared the mean syntenic distance of associating genes, versus the mean syntenic distance of abundant accessory gene pairs chosen at random. The average chromosome length across the data set is 6.2 Mbps; which, in addition to the chromosome being circular, means that the furthest away two genes could be from each other is ∼3.1 Mbps. The mean syntenic distance between randomly paired abundant accessory genes is bell-shaped that fits our expectation of randomly dispersed genes. In contrast, associating gene pairs more often share significant localization (fig. 2*a*); however, only 8.6% of all co-occurring gene pairs have a mean syntenic distance of <150 kbp. This suggests that a proportion of, but not all, gene–gene co-occurrence is due to colocalized genes.

In order to ask whether the colocalization patterns of gene pairs generalize to that of gene sets, we next considered gene associations in terms of their connected component (i.e., associating gene set; fig. 1*a*). We observe 41 gene sets (26%) that are composed of pairs of genes with a mean pairwise syntenic distance of ≤150 kbp (fig. 2*b*). We used PPanGGOLiN (Gautreau et al. 2020) to generate pangenome graphs of *Pseudomonas* spp*.* (supplementary fig. 6, Supplementary Material online) and the *P. aeruginosa* subset (fig. 2*c*) to visualize the genomic context of colocalized gene sets. For example, the *P. aeruginosa* pangenome graph includes a set of neighboring co-occurring genes associated with flagellar assembly (fig 2*c*, box 1). Interestingly, these genes are all encoded on the same strand, and this path in the pangenome graph bypasses a set of 16 genes, which also show homology to flagellar assembly genes (supplementary table 1, Supplementary Material online). A given genome may contain one but not both of these sets of genes, indicating possible redundancy of this function within the pangenome. We also observe gene sets that share very little physical linkage, such as a set of three unnamed genes involved in outer membrane permeability (fig. 2*c*, box 2; supplementary table 1, Supplementary Material online). Still, other gene sets have mixed levels of colocalization amongst their membership. For example, a subset of *P. aeruginosa* strains contains three neighboring genes (encoded on the same strand) that co-occur with a fourth gene sharing no physical linkage with the other three and encoded on the opposite strand (fig. 2*c*, box 3). These four genes likely co-occur because they all function as components of the methionine salvage pathway (supplementary fig. 7 and table 1, Supplementary Material online).

### Coincident Genes Share Functionality

**Fig. 3.**
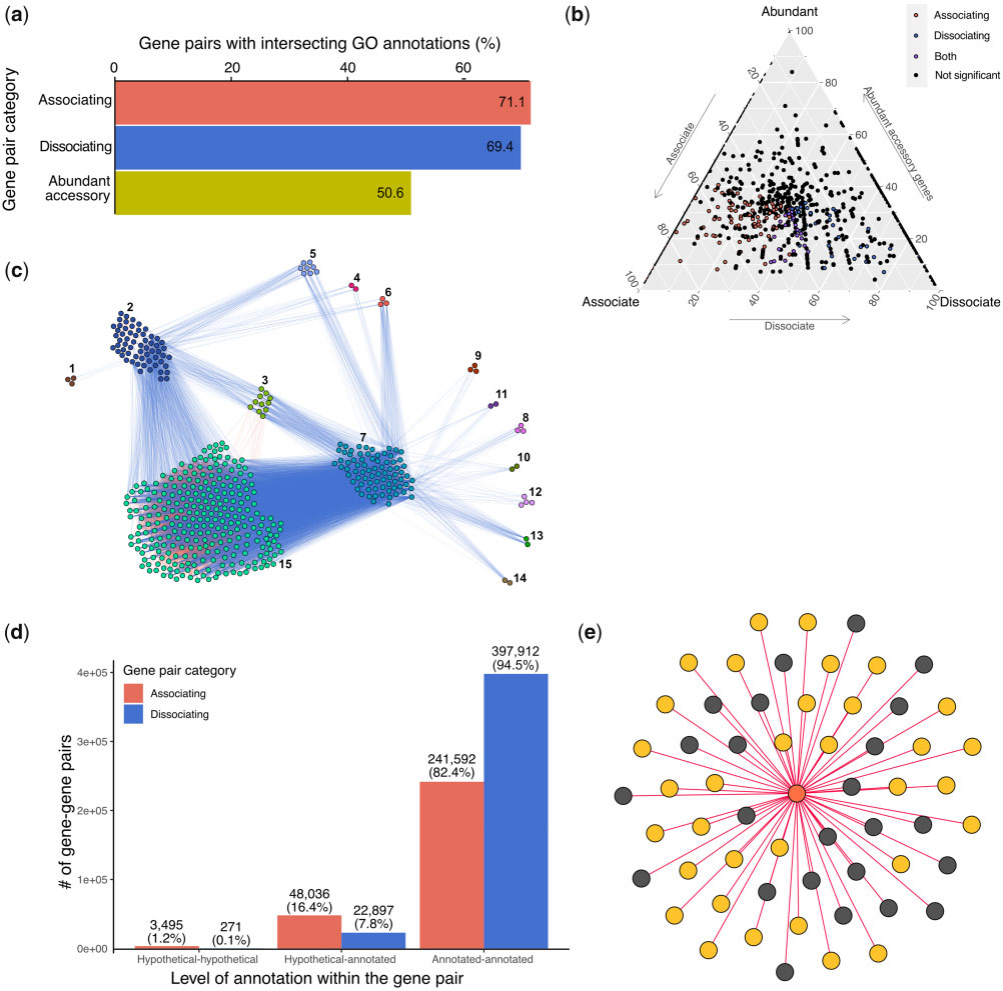
Coincident (associating and dissociating) gene pairs have more overlapping GO term annotations when compared with random gene pairs. (*a*) 71.1% of associating gene pairs share the same GO annotations compared with 50.6 (± 0.1)% of randomly paired genes. (*b*) A triangular plot of GO term annotation within coincident gene space. Each GO term is represented by a point whose location is determined by how frequently genes with that term are found in the associating, dissociating, and random gene pair categories. GO terms which are significantly overrepresented in any category are colored. (*c*) Coincident gene relationships for genes annotated with transmembrane transporter activity (GO:0022857). Edges are colored by the type of interaction (associating, red; dissociating, blue). A figure showing only the associating edges is provided in supplementary fig. 8*a*, Supplementary Material online. (*d*) The proportion of coincident gene pair relationships which exist between annotated and hypothetical genes. (*e*) A network of gene (node) association relationships (edges) depicting the associations of *ampC* (orange) with hypothetical (gray) and annotated (yellow) genes.

The association (or dissociation) of genes alone does not infer a biological interaction between them (i.e., correlation does not infer causation; Blanchet et al. 2020). If the accessory genome is influenced by selection, we could expect that coincident genes might be more likely to act together—for example, toward a shared functional goal—for the benefit of the host. Alternatively, genes might act together toward their own selection (e.g., in DNA secretion; Draghi and Turner 2006) or integrative conjugative elements (Wozniak and Waldor 2010). Using Gene ontology (GO) annotations as a proxy for gene functionality, we calculated the functional overlap of each coincident gene pair in comparison to randomly paired abundant accessory genes, indicating some structure in the accessory pangenome (fig. 3*a*). We identified a greater overlap in GO annotations between coincident gene pairs then randomly paired accessory genes. Specifically, 71.1% of associating and 69.4% of dissociating gene pairs shared GO annotations when compared with only 50.6 (±0.1)% of randomly paired accessory genes (fig. 3*a*). This indicates that coincident genes share function with each other more often than would be expected by chance. The percentage of shared GO annotations amongst associating genes increased to 74% when only nonsyntenic genes were considered (supplementary fig. 8, Supplementary Material online). Given these results, we calculated whether particular GO terms were more likely to share annotation in a coincident gene pair compared with the expected term-sharing frequency (fig. 3*b*). One hundred and fifty GO terms were found to be overrepresented in gene–gene associations, including pilus assembly (GO:0009297; *P* = 1.41e−05), type II protein secretion system complex (GO:0015627; *P* = 1.35e−08), and antibiotic biosynthetic process (GO:0017000; *P* = 4.84e−10) (fig. 3*b*, red points, supplementary table 2, Supplementary Material online). In contrast, 60 GO terms were overrepresented in dissociation relationships, including ATP-binding cassette transporter complex (GO:0043190; *P* = 4.96e−52), and drug transmembrane transport (GO:0006855; *P* = 2.16e−07) (fig. 3*b*, blue points, supplementary table 2, Supplementary Material online).

A subset of GO annotations were enriched in both associating and dissociating gene pairs (fig. 3*b*, purple points; supplementary table 2, Supplementary Material online). This appears counterintuitive, but may correspond to, for example, two multigene functional units that dissociate from one another but whose genes within the unit strongly associate with each other. For example, gene pairs annotated with transmembrane transporter activity (GO:0022857) were enriched in association (*P* = 8.39e−06) and dissociation gene relationships (*P* = 3.01e−28; fig. 3*c*). Although some genes formed independent co-occurring cliques or solitary dissociation patterns (not shown), the majority of genes clustered into groups of associating genes (supplementary fig. 9*a*, Supplementary Material online) that dissociated from each other (fig. 3*c*). Some of these cluster avoidance patterns appear to be largely due to species boundaries (e.g., clusters 7 and 15; supplementary fig. 9*b*, Supplementary Material online) but most are independent of phylogeny and syntenic relationships (supplementary fig. 9*b* and *c*, Supplementary Material online). Although many of these genes are hypothetical or only loosely annotated, there are, for example, genes for an efflux pump (Resistance-nodulation-division family transporters) in cluster 2 that dissociate from genes for a different efflux pump (glutathione-regulated potassium-efflux system protein, KefB) in cluster 3 (supplementary table 3, Supplementary Material online), indicating a possible example of functional redundancy or niche partitioning within this system. We also identify gene–gene association patterns between genes with known biological interactions such as bfmS and bfmR that form the BfmS/R two-component system (cluster 9; Cao et al. 2014) and cynS and cynT that are involved in cyanate decomposition (cluster 11; Guilloton et al. 1993; Luque-Almagro et al. 2008).

The above calculations of intersecting GO annotations rely on known gene information. Although *Pseudomonas* is a well-studied genus with well-annotated genomes, many of the identified coincident gene pairs involve interactions between hypothetical proteins or genes without a known GO association. 51,531 (17.6%) and 23,168 (7.9%) of the associating and dissociating gene pairs, respectively, involve at least one hypothetical gene (fig. 3*d*). Specifically, 95% of coincident gene pairs involving hypothetical genes are between hypothetical and annotated genes. Given our finding that many annotated coincident gene pairs share function, coincident relationships between hypothetical and annotated genes can help us generate hypotheses concerning the role these hypothetical proteins play in the *Pseudomonas* sp. pangenome. A subset of GO terms was found to be statistically more likely to be coincident with hypothetical genes when compared with the annotated coincident gene pairs (supplementary table 4, Supplementary Material online). For example, the “beta-lactamase activity” (*P* = 1.86e−06; GO:0008800) GO annotation was assigned to two genes that collectively associated with 120 annotated and 33 hypothetical genes. In particular, 42% of the genes that associate with an *ampC* homolog (most closely related to PDC-8; Rodríguez-Martínez et al. 2009) were annotated as hypothetical proteins, and only seven had a gene name annotation in ≥1 genome (fig. 3*e*, supplementary table 5, Supplementary Material online). This gene association cluster (including *ampC*) is present in ≥4 *Pseudomonas* species (4 named, 6 unnamed strains), and does not share considerable colocalization across the pangenome (supplementary fig. 10, Supplementary Material online). Similar investigations of the remaining hypothetically-annotated gene pairs may yield further hypotheses concerning the role of hypothetical proteins in this pangenome.

### Gene Co-occurrence Is Associated with Co-transcription and Protein–Protein Interactions

**Fig. 4.**
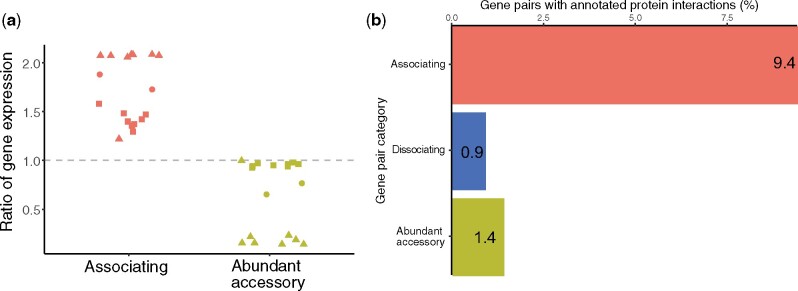
Associating genes are more likely to be cotranscribed. (*a*) The ratio of gene expression between associating gene pairs and random abundant accessory gene pairs. The ratio is calculated as the proportion of times that both genes in a gene pair are consistently cotranscribed across *P. aeruginosa* genomes versus the proportion of times that only one of the two genes is transcribed. Symbols represent different publicly available RNA-Seq experimental projects. (*b*) Protein–protein interaction pairs as compared with the STRING database indicate more interactions in the associating gene pairs compared with the dissociating and random gene–gene data. One hundred replicates of randomly paired genes were used to obtain a mean of 1.4 (±0.03)%.

Using publicly available RNA-Seq transcription data, we examined how often associating gene pairs were transcribed together compared with randomly paired accessory genes. Due to limitations on the availability of good quality publicly available gene transcription data, we restricted our analysis to *P. aeruginosa* (81 of 209 genomes). Across the *P. aeruginosa* pangenome, we calculated the frequencies with which a given gene pair was transcribed together compared with that of only one of the two genes in a pair. We report this ratio of gene expression, such that a ratio of 1.0 indicates that—across the *P. aeruginosa* pangenome—it is as likely to see both genes transcribed together as it is for only one of the pair to be transcribed (fig. 4*a*). Across samples and experiments, associating gene pairs were more often co-transcribed than were randomly paired abundant accessory genes (fig. 4*a*), indicating a possible shared function or interaction between these genes. This result holds when only nonsyntenic gene associations are considered (supplementary fig. 11, Supplementary Material online). Similar analyses of cotranscription could not be performed on the dissociating gene pairs as these pairs are not present within the same genomes.

Given the rate of co-transcription of associating genes, we asked how often coincident genes are involved in known protein–protein interactions. Using the STRING database (Szklarczyk et al. 2019), we first identified the number of protein–protein interactions between randomly paired accessory genes as 1.4 (±0.03)%. This percentage may seem low; however, we expect that documented protein–protein interactions are more likely to involve well-studied, abundant (likely core), house-keeping proteins, or those which share evolutionary histories with each other, which are precisely the genes that are excluded in our analyses of lineage-independent accessory genes. However, we identified protein–protein interactions between 9.4% of associating gene pairs (11.4% of all annotated associating pairs; Figure 4b). These data represent 2.5% of all known protein–protein interactions within *P. aeruginosa*; that is to say that—even when excluding core or vertically inherited genes—associating gene relationships recapitulates a percentage of all known protein interactions in this species. As expected, evidence of interactions between dissociating genes was identified at a rate less than randomly paired genes (fig. 4*b*).

## Discussion

We recently developed a novel method for the identification of coincident gene presence–absence patterns within pangenomes (Whelan et al. 2020). Although pangenome analyses are usually focused on a particular species, here we applied this approach to 209 publicly available *Pseudomonas* spp. genomes to ask whether a portion of pangenome gene content is determined by selective pressure. Across the data set, 86.7% of lineage-independent, abundant accessory genes consistently associated with, or dissociated from, at least one other gene in the pangenome. This represents a lot more genetic structure within the abundant accessory genome than we would expect if neutral processes were driving pangenome formation. We found that these gene pairs share functional annotations, are co-transcribed, and produce proteins that interact with each other more often than expected when compared with randomly paired abundant accessory genes. These findings were independent of whether the genes are lineage-dependent or are predominantly vertically transmitted and the association remained even when colocalized genes were excluded. The fact that we found statistically significant associations between nonsyntenic genes is strong evidence for selection because it identifies genes that share functionality despite being dispersed in the genome. Together, these data suggest that the assemblage of abundant accessory genes is structured in this pangenome is more likely explained by selection than by neutral processes. This work has implications for our understanding of prokaryote pangenomes as a whole.

However, we should be mindful of the limitations of this approach. For example, because of the nature of identifying coincident relationships, this analysis can only be conducted when a gene is present frequently enough across a data set. In this case, we used a threshold of >5% abundance, which equated to a focus on the 13,864 most abundant accessory genes. Further, gene–gene co-occurrence does not necessarily infer direct gene–gene interactions. Although such a high-throughput examination of gene–gene co-occurrence relationships in pangenomes may be rare (Kim and Price 2011; Cohen et al. 2012; Press et al. 2016), there is a century of literature on species–species co-occurrence patterns (Forbes 1907; Michael 1920; Diamond 1975; Connor and Simberloff 1979; Blanchet et al. 2020). In this research, it has been explicitly shown that in at least some cases, species–species co-occurrence does not necessarily imply species–species ecological interactions. In their recent Perspectives article, Blanchet et al. (2020) present seven arguments for why ecological interaction should not be assumed from co-occurrence data. Although some of these arguments are species-specific, many apply to gene–gene data as well. For example, the authors argue that in some cases, species occurrences depend on the environment, and what appears as a species–species co-occurrence pattern may actually be an indirect interaction of both species with their environment (Blanchet et al. 2020); similarly, *geneA* and *geneB* may co-occur due to a preference for a shared abiotic factor—environment, nutrient, metabolite, etc.—instead of indicating a direct gene-to-gene interaction. We suggest that further *in vitro* investigations of gene pairs could help clarify these levels of interactions. Further, the methodology used here—the identification of coincident gene relationships based on statistically similar or dissimilar gene presence/absence patterns—will not identify all associations in the pangenome. For example, asymmetrical dependencies will have been missed; in the case where *geneA* relies on *geneB* for an interaction but not vice versa, we would expect to see *geneA* present only in the presence of *geneB*, but that *geneB* could be present without *geneA* in a given genome. So-called “event horizon genes” or those genes whose presence “leads the way” for the introduction of many other genes (McInerney et al. 2020), will also not be identified by use of the Coinfinder software. Because these gene–gene patterns are hard to distinguish from random presence/absence patterns, their influence on the structure of the pangenome will be harder to determine.

With this caveat in mind, we sought to provide evidence for the possibility that a sizable subset of the gene–gene associations within the abundant accessory genes of the *Pseudomonas* pangenome may be due to direct interactions. The fact that many associating gene pairs tend to neighbor each other indicates this potential. Neighboring genes often assemble into sets of co-transcribed genes that either physically interact to form protein complexes (e.g., *manXYZ*; Erni et al. 1987) or act as part of a shared metabolic pathway (e.g., the *lac* operon; Jacob and Monod 1961). However, many coincident genes that were not colocalized had overlapping functionality. This is not too surprising given previous evidence of coselection of genes (Cui et al. 2020) and single-nucleotide polymorphisms ( Cui et al. 2020; Pensar et al. 2019) across disperse loci. These genes could still directly interact, although could also indicate a response to a shared abiotic factor (e.g., genes present in response to a particular environmental niche). On the other hand, genes with shared functionalities that actively avoid each other would seem to suggest a more directed type of interaction. Further, strain-level variation in gene essentiality can also contribute to gene–gene association patterns. For example, it has been recently shown that the horizontal acquisition of specific genes in *E. coli* can make a gene that was already present in the genome as a nonessential accessory gene become newly essential (Rousset et al. 2021). Either way, evidence for interactions at the protein level clearly indicates direct gene–gene interactions in the accessory pangenome.

One of the inspirations for this work was the recent suggestion that one way of better elucidating whether the pangenome is evolving neutrally or adaptively was to focus on the gene as the evolutionary unit (Shapiro 2017). Examining gene–gene relationships, as we have done here, is not the only gene-focused approach to understanding the evolutionary pressures present on prokaryote pangenomes. For example, analyses could be conducted to determine whether accessory genes are under selective pressures. Further, gene knockout and long-term evolutionary experiments could be combined to determine the effect of individual genes on the pangenome. Similarly, we could adapt the unit of observation to think about groups of genes; for example, we could ask whether operons, or genes which comprise multi-subunit proteins form coincident relationships. We propose these results concerning gene–gene coincident relationships as one line of evidence for testing hypotheses of selective pressures on the accessory genome. We encourage further work in these areas to be contributed to this debate.

We focused our analysis on *Pseudomonas* spp. due to its diverse, well-studied pangenome (Kung et al. 2010; Mosquera-Rendón et al. 2016; Udaondo et al. 2016; Dillon et al. 2019; Freschi et al. 2019), well-annotated genomes (Winsor et al. 2016), and generalizability to other prokaryotic open pangenomes in terms of core-to-accessory gene ratios, and multiple environmental niches. Our results suggest genetic structure within this pangenome, and we hope that additional research, using different methodologies and pangenomes, will help further these findings.

## Materials and Methods

### Sequence Acquisition and Pangenome Analysis

Genome annotations were retrieved from pseudomonas.com in GFF3 format (Winsor et al. 2016) on March 1, 2019 and include 209 complete genome assemblies. Despite the availability of thousands of draft genomes, we restricted our study to completely assembled and curated strains, due to recent work suggesting that the quality of genome assembly can greatly impact predicted pangenome quality and size (Denton et al. 2014). Genes were clustered into gene families using Roary 3.12.0 (Page et al. 2015) with a 70% BLASTP percentage identity cutoff. Definitions of core (90%≤x≤100%), soft core (89%≤x < 90%), shell (15%≤x < 89%), and cloud (x < 15%) genes are as in Roary. All core genes (present in ≥90% of *Pseudomonas* genomes) were individually aligned using MAFFT v7.310 (Katoh and Standley 2013), the alignments concatenated, and curated using Gblocks (Castresana 2000; parameters as in Creevey et al. [2011]), specifically allow gap positions = half, minimum length of block = 2). A core gene phylogeny was constructed from this curated and concatenated alignment using IQ-TREE (Nguyen et al. 2015) using the GTR+I + G substitution model (as justified in Abadi et al. [2019]) and midpoint rooted. A total of 19 genome annotations contained plasmids, which were not considered in these analyses.

### Evaluation of Gene Coincident Relationships

Coincident relationships between gene pairs were determined using Coinfinder (Whelan et al. 2020). Briefly, for each pair of genes in the input accessory genome, Coinfinder examines their presence/absence patterns to determine if they represent a coincident relationship (i.e., if they co-occur or avoid each other across the pangenome more often than expected by chance). Statistically significant coincident gene pairs were determined by Coinfinder *via* a Bonferroni-corrected binomial exact test statistic, and the lineage dependence of each gene was calculated using a previously established phylogenetic measure of binary traits (D; Fritz and Purvis 2010). Coinfinder was run with upper- and lower-filtering gene abundance thresholds of 90% and 5%, respectfully. A threshold of D≥−0.4 was used based on the frequency of genes, their distribution across species in the core gene phylogeny, and the distribution of counts of coincident gene pairs (supplementary fig. 3, Supplementary Material online). The resulting associating and dissociating networks were visualized using Gephi (Bastian et al. 2009). Hub genes were defined as those with more gene–gene relationships than 1.5 times the upper interquartile range.

In order to determine whether coincident gene pairs were more likely to share functional annotations, gene expression patterns, or protein–protein interactions (see below), we compared these results against the null model by generating random abundant accessory gene pairs. To do so, accessory genes that were included in the Coinfinder analysis (i.e., were between 5% and 90% abundance with D≥−0.4) were paired at random to match the mean number of associating/dissociating gene pairs (*n* = 357,102) in 100 replicates (herein referred to as random abundant accessory gene pairs). This was accomplished by creating a list of all possible paired combinations of abundant accessory gene pairs and creating *n* = 100 random permutations of the list to a length of 357,102. The specific use of these random abundant accessory gene pairs is outlined in the following Materials and Methods sections.

### Gene Colocalization and Pangenome Structure Analysis

The physical linkage between genes in a gene pair was determined both for associating, and for random abundant accessory gene pairs. For a given gene pair, the physical distance between *geneA* and *geneB* was calculated for each genome for which both *geneA* and *geneB* reside. (For this reason, syntenic distance information could not be calculated for dissociating gene pairs.) From these *geneA–geneB* distances for each genome, a mean syntenic distance was computed and plotted. In analyses of nonsyntenic genes, only those gene pairs separated by a mean syntenic distance of ≥150 kbp were considered.

A pangenome graph was created with PPanGGOLiN (Gautreau et al. 2020). In order to maintain consistency with the gene cluster information used throughout this study, PPanGGOLiN was provided with the gene clusters as determined by Roary. A Python script was used to redefine nodes in the pangenome graph to remain consistent with the definitions of core, soft core, shell, and cloud that are used by Roary. The nodes of the resulting graph were recolored to represent the associating gene sets as determined by Coinfinder. The network was visualized in Gephi (Bastian et al. 2009). KEGG was used to investigate metabolic pathways (Kanehisa and Goto 2000).

### Functional Annotations of Coincident Genes

GO term annotations for each of the 209 genomes were collected from pseudomonas.com on March 22, 2019. A minimum of one matching GO term annotation was necessary to consider a gene pair as having overlapping function. Overlapping annotations were determined by examining only those gene pairs for which both genes had a GO term annotation. After removing gene pairs for which GO term annotations were missing for one or both genes, a total of 246,637 (84.1%) associating, and 379,439 (90.11%) dissociating gene pairs remained. These were compared with 100 replicates of randomly paired abundant accessory genes as described above. Bonferroni-corrected binomial tests (computed in R; R Core Team 2017) were used to determine which GO terms were under- or over-represented in the coincident gene pairs when compared with the random abundant accessory gene pairs.

Separately, GO terms that were significantly associated with genes of hypothetical function were determined. Genes were defined as hypothetical if every instance of the gene across all genomes in which it was found were annotated as “hypothetical protein.” Bonferroni-corrected binomial tests were used to determine GO terms over-represented in gene pairs involving an annotated and hypothetical gene. Subnetworks of specific gene–gene interaction pairs were displayed using Gephi (Bastian et al. 2009).

### Gene Expression Analysis

Short read archive transcript data from the following *P. aeruginosa* RNA-Seq experiments (paired-end reads with a range of 4,450,537–41,817,822 reads per sample) were used to test co-transcription levels of gene–gene pairs: SRP163899 (*n* = 2 samples), SRP215630 (*n* = 9), and SRP191772 (*n* = 8; Zhang et al. 2019). The reads from each RNA-Seq sample were mapped using Bowtie2 (Langmead and Salzberg 2012) to the gene content of the *P. aeruginosa* genomes in the dataset (*n* = 81). In a given genome, a gene was considered transcribed if ≥85% of the gene’s length was covered by ≥2 reads. Across the dataset, a gene cluster was considered transcribed if it was transcribed in ≥75% of the genomes in which it was present. The ratio of gene expression is the ratio of gene cluster pairs that are co-transcribed versus those in which only one of the two genes were transcribed. Therefore, a ratio of 1.0 would mean that, across all *P. aeruginosa* genomes, paired genes are just as likely to be co-transcribed as for exclusively one of the two genes to be transcribed; a ratio of 2.0 would mean that paired genes are twice as likely to be transcribed together across the pangenome.

### Protein Interaction Analysis

The STRING database (Szklarczyk et al. 2019) was used to identify whether the protein products of associating, dissociating, and random abundant accessory gene pairs interact with each other. The protein network data and associated FASTA sequences for *P. aeruginosa* were obtained from https://string-db.org (accessed May 9 2019). The FASTA sequences for the proteins in this network were assembled into a BLAST database to map homologous gene clusters to the IDs in the STRING protein network, with the criteria of ≥85% coverage and ≥90% sequence identity. Calculations of the coincident gene pairs were compared with 100 replicates of randomly paired abundant accessory gene pairs as described above.

## Supplementary Material

Supplementary data are available at *Molecular Biology and Evolution* online.

## Supplementary Material

msab139_Supplementary_Data

## References

[msab139-B1] Abadi S, Azouri D, Pupko T, Mayrose I. 2019. Model selection may not be a mandatory step for phylogeny reconstruction. Nat Commun. 10(1):934. 30804347 10.1038/s41467-019-08822-w PMC6389923

[msab139-B2] Andreani NA, Hesse E, Vos M. 2017. Prokaryote genome fluidity is dependent on effective population size. ISME J. 11(7):1719–1721. 28362722 10.1038/ismej.2017.36 PMC5520154

[msab139-B3] Azarian T, Huang I-T, Hanage WP. 2020. Structure and dynamics of bacterial populations: pangenome ecology. In: Tettelin H, Medini D, editors. The pangenome: diversity, dynamics and evolution of genomes. Cham: Springer International Publishing. p. 115–128. 32633912

[msab139-B4] Bastian M, Heymann S, Jacomy M. 2009. Gephi: an open source software for exploring and manipulating networks. International AAAI Conference on Weblogs and Social Media. Available from: https://www.aaai.org/ocs/index.php/ICWSM/09/paper/view/154

[msab139-B5] Blanchet FG, Cazelles K, Gravel D. 2020. Co-occurrence is not evidence of ecological interactions. Ecol Lett. 23(7):1050–1063. 32429003 10.1111/ele.13525

[msab139-B6] Bobay L-M, Ochman H. 2018. Factors driving effective population size and pan-genome evolution in bacteria. BMC Evol Biol. 18(1):153. 30314447 10.1186/s12862-018-1272-4 PMC6186134

[msab139-B7] Cao Q, Wang Y, Chen F, Xia Y, Lou J, Zhang X, Yang N, Sun X, Zhang Q, Zhuo C, et al 2014. A novel signal transduction pathway that modulates rhl quorum sensing and bacterial virulence in *Pseudomonas aeruginosa*. PLoS Pathog. 10(8):e1004340. 25166864 10.1371/journal.ppat.1004340 PMC4148453

[msab139-B8] Castresana J. 2000. Selection of conserved blocks from multiple alignments for their use in phylogenetic analysis. Mol Biol Evol. 17(4):540–552. 10742046 10.1093/oxfordjournals.molbev.a026334

[msab139-B9] Cohen O, Ashkenazy H, Burstein D, Pupko T. 2012. Uncovering the co-evolutionary network among prokaryotic genes. Bioinformatics 28(18):i389–i394. 22962457 10.1093/bioinformatics/bts396 PMC3436823

[msab139-B10] Connor EF, Simberloff D. 1979. The assembly of species communities: chance or competition? Ecology 60(6):1132.

[msab139-B11] Creevey CJ, Doerks T, Fitzpatrick DA, Raes J, Bork P. 2011. Universally distributed single-copy genes indicate a constant rate of horizontal transfer. PLoS One 6(8):e22099. 21850220 10.1371/journal.pone.0022099 PMC3151239

[msab139-B12] Cui Y, Yang C, Qiu H, Wang H, Yang R, Falush D. 2020. The landscape of coadaptation in *Vibrio parahaemolyticus*. *eLife* 9:e54136. 10.7554/eLife.54136 PMC7101233 32195663

[msab139-B13] Decano AG, Downing T. 2019. An *Escherichia coli* ST131 pangenome atlas reveals population structure and evolution across 4,071 isolates. Sci Rep. 9(1):17394. 10.1038/s41598-019-54004-5 PMC6874702 31758048

[msab139-B14] Denton JF, Lugo-Martinez J, Tucker AE, Schrider DR, Warren WC, Hahn MW. 2014. Extensive error in the number of genes inferred from draft genome assemblies. PLoS Comput Biol. 10(12):e1003998. 25474019 10.1371/journal.pcbi.1003998 PMC4256071

[msab139-B15] Deutscher J, Francke C, Postma PW. 2006. How phosphotransferase system-related protein phosphorylation regulates carbohydrate metabolism in bacteria. Microbiol Mol Biol Rev. 70(4):939–1031. 17158705 10.1128/MMBR.00024-06 PMC1698508

[msab139-B16] Diamond J. 1975. Assembly of species communities. In: Diamond J, Cody M, editors. Ecology and evolution of communities. Boston:Harvard University Press. p. 342–344.

[msab139-B17] Dillon MM, Thakur S, Almeida RN, Wang PW, Weir BS, Guttman DS. 2019. Recombination of ecologically and evolutionarily significant loci maintains genetic cohesion in the *Pseudomonas syringae* species complex. Genome Biol. 20:3. 30606234 10.1186/s13059-018-1606-y PMC6317194

[msab139-B18] Ding W, Baumdicker F, Neher RA. 2018. panX: pan-genome analysis and exploration. Nucleic Acids Res. 46(1):e5. 29077859 10.1093/nar/gkx977 PMC5758898

[msab139-B19] Draghi JA, Turner PE. 2006. DNA secretion and gene-level selection in bacteria. Microbiology 152(9):2683–2688. 16946263 10.1099/mic.0.29013-0

[msab139-B20] Erni B, Zanolari B, Kocher HP. 1987. The mannose permease of *Escherichia coli* consists of three different proteins. J Biol Chem. 262(11):5238–5247. 2951378

[msab139-B21] Forbes SA. 1907. On the local distribution of certain Illinois fishes: an essay in statistical ecology. INHS Bull. 7(1–10):273–297.

[msab139-B22] Freschi L, Vincent AT, Jeukens J, Emond-Rheault JG, Kukavica-Ibrulj I, Dupont MJ, Charette SJ, Boyle B, Levesque RC. 2019. The *Pseudomonas aeruginosa* pan-genome provides new insights on its population structure, horizontal gene transfer, and pathogenicity. Genome Biol Evol. 11(1):109–120. 30496396 10.1093/gbe/evy259 PMC6328365

[msab139-B23] Fritz SA, Purvis A. 2010. Selectivity in mammalian extinction risk and threat types: a new measure of phylogenetic signal strength in binary traits. Conserv Biol. 24(4):1042–1051. 20184650 10.1111/j.1523-1739.2010.01455.x

[msab139-B24] Gautreau G, Bazin A, Gachet M, Planel R, Burlot L, Dubois M, Perrin A, Médigue C, Calteau A, Cruveiller S, et al 2020. PPanGGOLiN: depicting microbial diversity via a partitioned pangenome graph. PLoS Comput Biol. 16(3):e1007732. 32191703 10.1371/journal.pcbi.1007732 PMC7108747

[msab139-B25] Goyal A. 2018. Metabolic adaptations underlying genome flexibility in prokaryotes. PLoS Genet. 14(10):e1007763. 30372443 10.1371/journal.pgen.1007763 PMC6224172

[msab139-B26] Guilloton MB, Lamblin AF, Kozliak EI, Gerami-Nejad M, Tu C, Silverman D, Anderson PM, Fuchs JA. 1993. A physiological role for cyanate-induced carbonic anhydrase in *Escherichia coli*. J Bacteriol. 10.1128/jb.175.5.1443-1451.1993 PMC193231 8444806

[msab139-B27] Hiller NL, Sá-Leão R. 2018. Puzzling over the pneumococcal pangenome. Front Microbiol. 9:2580. 30425695 10.3389/fmicb.2018.02580 PMC6218428

[msab139-B28] Jacob F, Monod J. 1961. Genetic regulatory mechanisms in the synthesis of proteins. *J Mol Biol*. 3:318–356 10.1016/s0022-2836(61)80072-7 13718526

[msab139-B29] Kanehisa M, Goto S. 2000. KEGG: Kyoto Encyclopedia of Genes and Genomes. *Nucleic Acids Res*. 28(1):27–30. 10.1093/nar/28.1.27 PMC102409 10592173

[msab139-B30] Katoh K, Standley DM. 2013. MAFFT multiple sequence alignment software version 7: improvements in performance and usability. Mol Biol Evol. 30(4):772–780. 23329690 10.1093/molbev/mst010 PMC3603318

[msab139-B31] Kim P-J, Price ND. 2011. Genetic co-occurrence network across sequenced microbes. PLoS Comput Biol. 7(12):e1002340. 22219725 10.1371/journal.pcbi.1002340 PMC3248385

[msab139-B32] Klockgether J, Munder A, Neugebauer J, Davenport CF, Stanke F, Larbig KD, Heeb S, Schöck U, Pohl TM, Wiehlmann L, et al 2010. Genome diversity of *Pseudomonas aeruginosa* PAO1 laboratory strains. J Bacteriol. 192(4):1113–1121. 20023018 10.1128/JB.01515-09 PMC2812968

[msab139-B33] Kung VL, Ozer EA, Hauser AR. 2010. The accessory genome of *Pseudomonas aeruginosa*. Microbiol Mol Biol Rev. 74(4):621–641. 21119020 10.1128/MMBR.00027-10 PMC3008168

[msab139-B34] Langmead B, Salzberg SL. 2012. Fast gapped-read alignment with Bowtie 2. Nat Methods. 9(4):357–359. 22388286 10.1038/nmeth.1923 PMC3322381

[msab139-B35] Luque-Almagro VM, Huertas M-J, Sáez LP, Luque-Romero MM, Moreno-Vivián C, Castillo F, Roldán MD, Blasco R, 2008. Characterization of the *Pseudomonas pseudoalcaligenes* CECT5344 cyanase, an enzyme that is not essential for cyanide assimilation. Appl Environ Microbiol. 74(20):6280–6288. 18708510 10.1128/AEM.00916-08 PMC2570302

[msab139-B36] McInerney JO, McNally A, O’Connell MJ. 2017a. Reply to ‘The population genetics of pangenomes’. Nat Microbiol. 2(12):1575. 29176701 10.1038/s41564-017-0068-4

[msab139-B37] McInerney JO, McNally A, O’Connell MJ. 2017b. Why prokaryotes have pangenomes. Nat Microbiol. 2(4):17040. 28350002 10.1038/nmicrobiol.2017.40

[msab139-B38] McInerney JO, Whelan FJ, Domingo-Sananes MR, McNally A, O’Connell MJ. 2020. Pangenomes and selection: the public goods hypothesis. In: Tettelin H, Medini D, editors. The pangenome: diversity, dynamics and evolution of genomes. Cham: Springer. p.151–167. 32633920

[msab139-B39] Michael EL. 1920. Marine ecology and the coefficient of association: a plea in behalf of quantitative biology. J Ecol. 8(1):54.

[msab139-B40] Mosquera-Rendón J, Rada-Bravo AM, Cárdenas-Brito S, Corredor M, Restrepo-Pineda E, Benítez-Páez A. 2016. Pangenome-wide and molecular evolution analyses of the *Pseudomonas aeruginosa* species. BMC Genomics. 17(1):45. 10.1186/s12864-016-2364-4 PMC4710005 26754847

[msab139-B41] Nguyen L-T, Schmidt HA, von Haeseler A, Minh BQ. 2015. IQ-TREE: A fast and effective stochastic algorithm for estimating maximum-likelihood phylogenies. Mol Biol Evol. 32(1):268–274. 25371430 10.1093/molbev/msu300 PMC4271533

[msab139-B42] Page AJ, Cummins CA, Hunt M, Wong VK, Reuter S, Holden MT, Fookes M, Falush D, Keane JA, Parkhill J. 2015. Roary: rapid large-scale prokaryote pan genome analysis. Bioinformatics 31(22):3691–3693. 26198102 10.1093/bioinformatics/btv421 PMC4817141

[msab139-B43] Pensar J, Puranen S, Arnold B, MacAlasdair N, Kuronen J, Tonkin-Hill G, Pesonen M, Xu Y, Sipola A, Sánchez-Busó L, et al 2019. Genome-wide epistasis and co-selection study using mutual information. Nucleic Acids Res. 47(18):e112. 31361894 10.1093/nar/gkz656 PMC6765119

[msab139-B44] Press MO, Queitsch C, Borenstein E. 2016. Evolutionary assembly patterns of prokaryotic genomes. Genome Res. 26(6):826–833. 27197212 10.1101/gr.200097.115 PMC4889971

[msab139-B45] R Core Team. 2017. R: a language and environment for statistical computing. Vienna, Austria: R Foundation for Statistical Computing.

[msab139-B46] Rodríguez-Martínez J-M, Poirel L, Nordmann P, 2009. Extended-spectrum cephalosporinases in *Pseudomonas aeruginosa*. Antimicrob Agents Chemother. 53(5):1766–1771. 19258272 10.1128/AAC.01410-08 PMC2681535

[msab139-B47] Rousset F, Cabezas-Caballero J, Piastra-Facon F, Fernández-Rodríguez J, Clermont O, Denamur E, Rocha EP, Bikard D. 2021. The impact of genetic diversity on gene essentiality within the *Escherichia coli* species. Nat Microbiol. 6:301–312. 33462433 10.1038/s41564-020-00839-y

[msab139-B48] Shapiro BJ. 2017. The population genetics of pangenomes. Nat Microbiol. 2(12):1574. 29176697 10.1038/s41564-017-0066-6

[msab139-B49] Stanier RY, Palleroni NJ, Doudoroff M. 1966. The aerobic pseudomonads: a taxonomic study. J Gen Microbiol. 10.1099/00221287-43-2-159 5963505

[msab139-B50] Szklarczyk D, Gable AL, Lyon D, Junge A, Wyder S, Huerta-Cepas J, Simonovic M, Doncheva NT, Morris JH, Bork P, et al 2019. STRING v11: protein-protein association networks with increased coverage, supporting functional discovery in genome-wide experimental datasets. Nucleic Acids Res. 47(D1):D607–D613. 30476243 10.1093/nar/gky1131 PMC6323986

[msab139-B51] Tettelin H, Masignani V, Cieslewicz MJ, Donati C, Medini D, Ward NL, Angiuoli SV, Crabtree J, Jones AL, Durkin AS, et al 2005. Genome analysis of multiple pathogenic isolates of *Streptococcus agalactiae*: implications for the microbial “pan-genome”. Proc Natl Acad Sci U S A. 102(39):13950–13955. 16172379 10.1073/pnas.0506758102 PMC1216834

[msab139-B52] Udaondo Z, Molina L, Segura A, Duque E, Ramos JL. 2016. Analysis of the core genome and pangenome of *Pseudomonas putida*. Environ Microbiol. 18(10):3268–3283. 26261031 10.1111/1462-2920.13015

[msab139-B53] Whelan FJ, Rusilowicz M, McInerney JO. 2020. Coinfinder: detecting significant associations and dissociations in pangenomes. Microb Genom. 6(3):e000338. 10.1099/mgen.0.000338 PMC7200068 32100706

[msab139-B54] Winsor GL, Griffiths EJ, Lo R, Dhillon BK, Shay JA, Brinkman F. 2016. Enhanced annotations and features for comparing thousands of Pseudomonas genomes in the Pseudomonas genome database. Nucleic Acids Res. 44(D1):D646–D653. 26578582 10.1093/nar/gkv1227 PMC4702867

[msab139-B55] Wozniak RA, Waldor MK. 2010. Integrative and conjugative elements: mosaic mobile genetic elements enabling dynamic lateral gene flow. *Nat Rev Microbiol*. 8(8):552–563. 10.1038/nrmicro2382 20601965

[msab139-B56] Wu H, Wang D, Gao F. 2021. Toward a high-quality pan-genome landscape of *Bacillus subtilis* by removal of confounding strains. Brief Bioinform. 22(2):1951–1971. 32065216 10.1093/bib/bbaa013

[msab139-B57] Zhang Y, Zhou CM, Pu Q, Wu Q, Tan S, Shao X, Zhang W, Xie Y, Li R, Yu XJ, et al 2019. *Pseudomonas aeruginosa* regulatory protein AnvM controls pathogenicity in anaerobic environments and impacts host defense. mBio. 10(4):e01362–19. 31337721 10.1128/mBio.01362-19 PMC6650552

