# Supplementary material for: Evidence for Selection in the Abundant Accessory Gene Content of a Prokaryote Pangenome
Source: Mol Biol Evol. 2021 May 8;38(9):3697–708. doi: 10.1093/molbev/msab139 (PMC8382901; doi:10.1093/molbev/msab139)
Supplement: msab139_Supplementary_Data [file msab139_supplementary_data.zip › PseudoPan_supplemental.pdf]

Supplementary Information

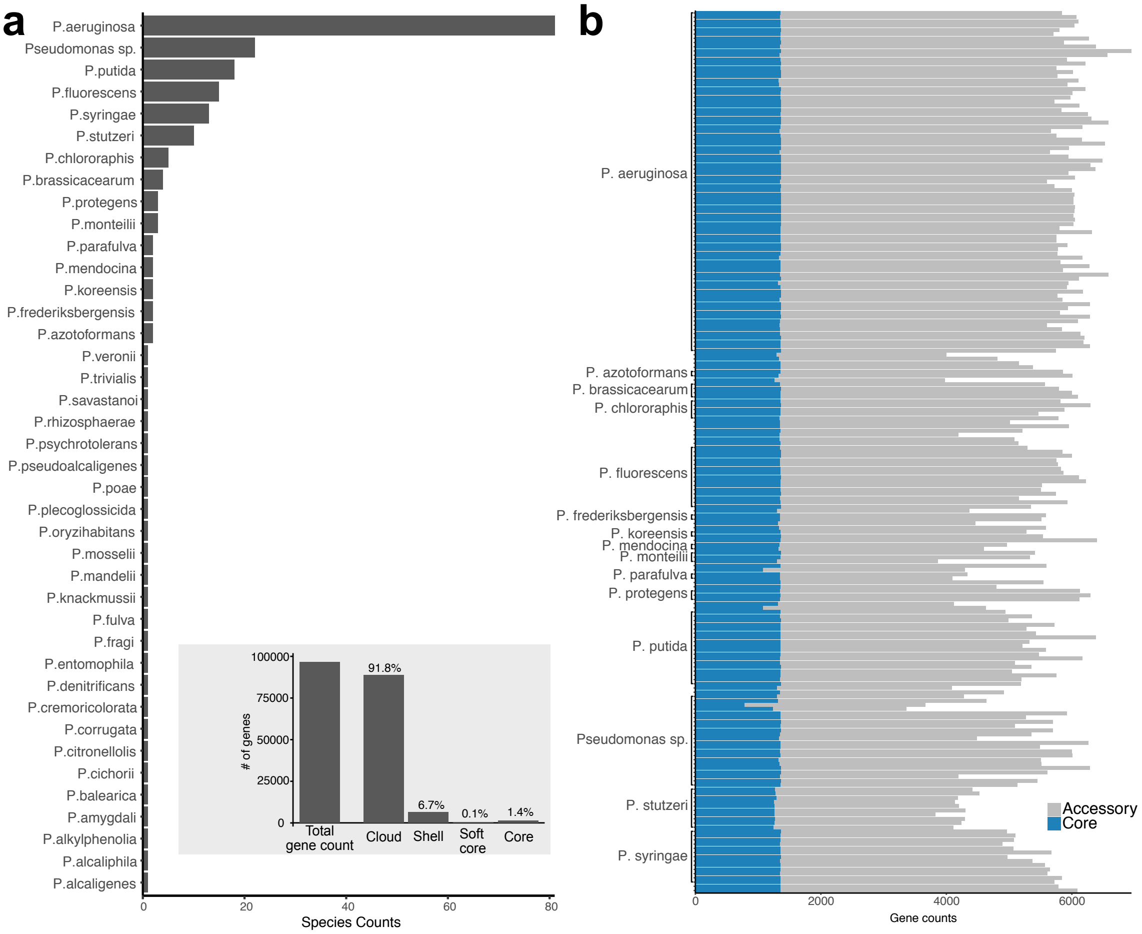

Supplementary Figure 1: **Distribution of *Pseudomonas* species and genes in the dataset. a.** The number of genomes per species present in the dataset (n=209). **a, inset.** The total gene cluster counts (n=96,694) across all strains broken down into the following categories: Cloud genes (present in 0% $\leq$ strains $<$ 15%), Shell genes (15% $\leq$ strains $<$ 89%), Soft core genes (89% $\leq$ strains $<$ 90%), and Core genes (90% $\leq$ strains $\leq$ 100%). Collectively, cloud, shell, and soft core genes are referred to as accessory genes. **b.** The distribution of core and accessory genes across all strains.

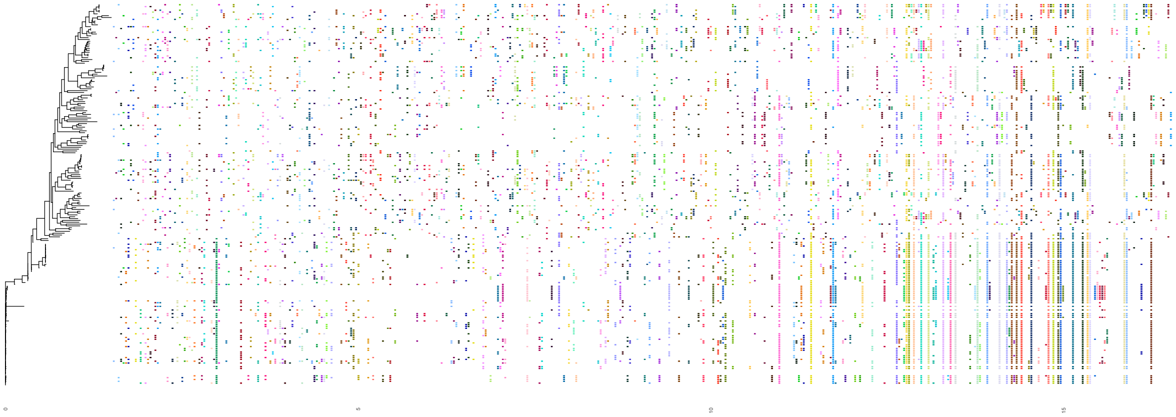

Supplementary Figure 2: **Mean presence of the genes comprising each connected component in the association dataset.** Each connected component (i.e. set of genes which associate with each other) is well dispersed across the core gene phylogeny indicating that vertically inherited, or lineage-dependent genes, have been successfully culled from the dataset. Each connected component is indicated by a single, randomly coloured column in the heatmap.

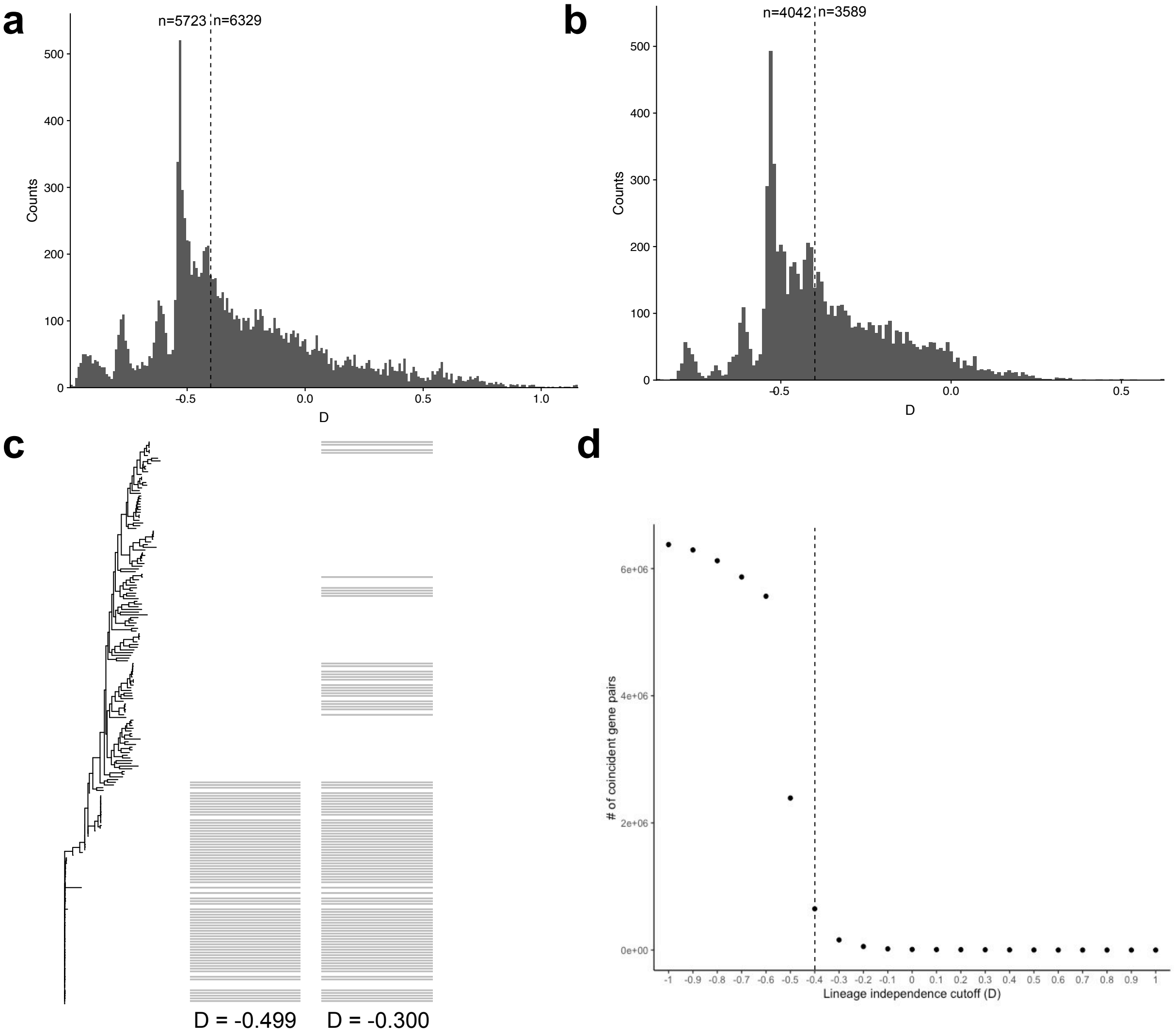

Supplementary Figure 3: **D-value distribution across the nodes found to have significant co-occurring (a) and avoidance (b) relationships in the complete genome dataset (n=209).** The D-value indicates the amount of lineage-independence of a particular gene compared to a phylogeny. Based on the counts and distribution of genes across the core gene phylogeny, a D-value cutoff of -0.4 was chosen, resulting in the inclusion of 6329 and 3589 gene clusters (i.e. nodes), respectively, in the co-occurrence and avoidance datasets. **c.** An example of the distribution of 2 genes across the core gene phylogeny with D values on either side of the chosen threshold. **d.** The distribution of counts of coincident gene pairs identified by Coinfinder at various cutoffs of lineage-independence. The vertical dashed line indicates the cutoff used in this analysis.

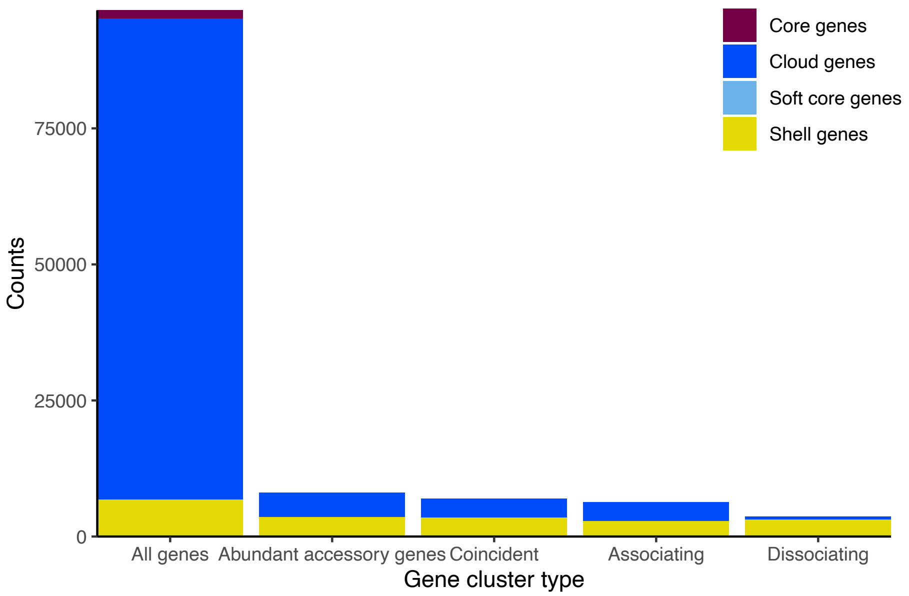

Supplementary Figure 4: **A non-log transformed version of the gene counts per category of gene cluster type.** A continuous representation of the counts shown in **Figure 1c**.

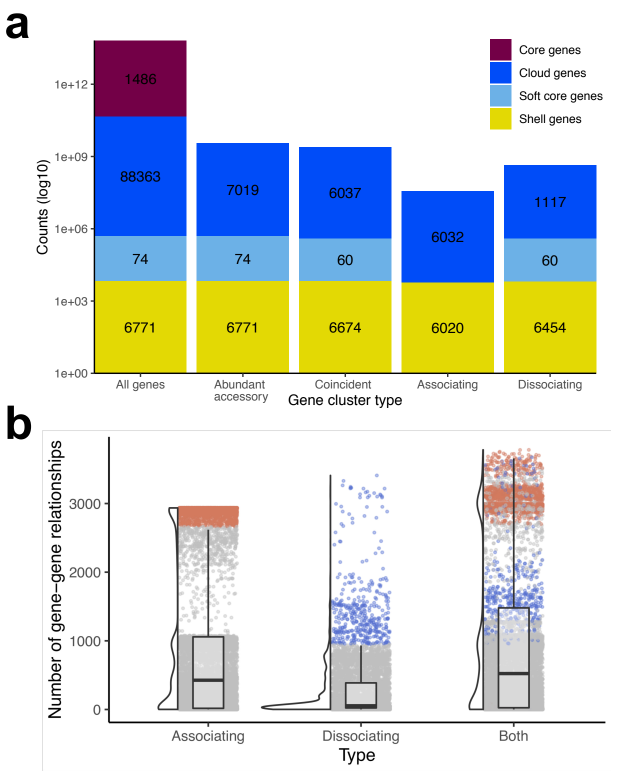

Supplementary Figure 5: **Coincident gene relationships between lineage-dependent and -independent genes. a.** Similar patterns of coincident gene relationships are seen when lineage-dependent genes are included when compared to counts of only lineage-independent genes (**Figure 1c**). **b.** However, the distribution of relationships per gene differs when lineage-dependent genes are included, including an increase in the number of hub genes when compared to those identified between lineage-independent genes (**Figure 1e**). Boxplots display the first and third quartiles, with a horizontal line to indicate the median, and whiskers extend to 1.5 times the interquartile range.

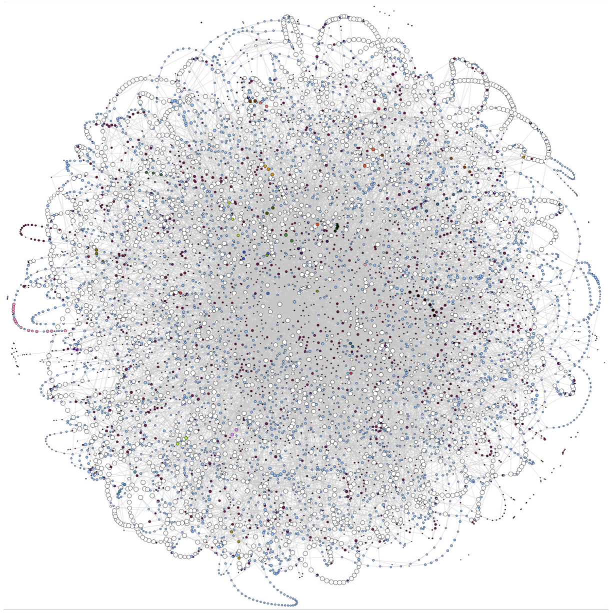

Supplementary Figure 6: **The graph of the *Pseudomonas sp.* pangenome**. The pangenome graph of the 209 *Pseudomonas sp.* genomes as determined by PPanGGOLiN. Each gene cluster (node) is coloured by its connected component with the same colour scheme used in **Figure 1a**. For visibility, cloud genes are not shown.

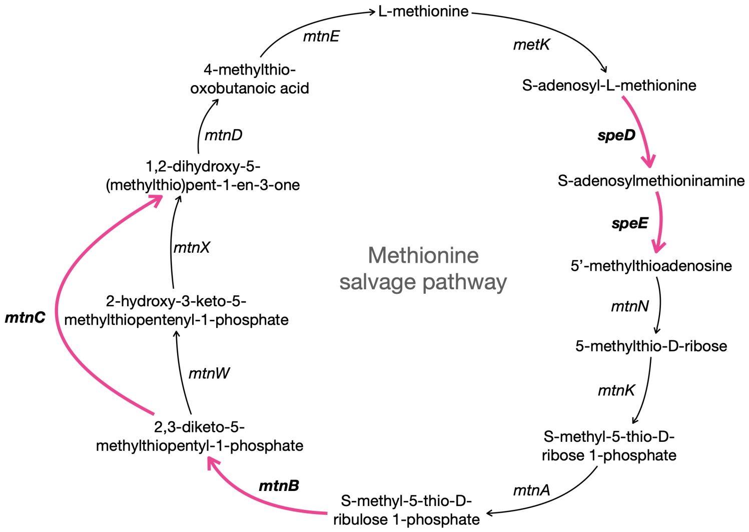

Supplementary Figure 7: **The location of the co-occurring gene set within the simplified pathway of methionine salvage.** Homologs of the gene clusters in question are bolded and the steps they are involved with coloured. These 4 genes were found to significantly co-occur but not all genes within the gene set are co-localized on the genome. This visual of select reactions and metabolites of the methionine salvage pathway is based off of information obtained from KEGG.

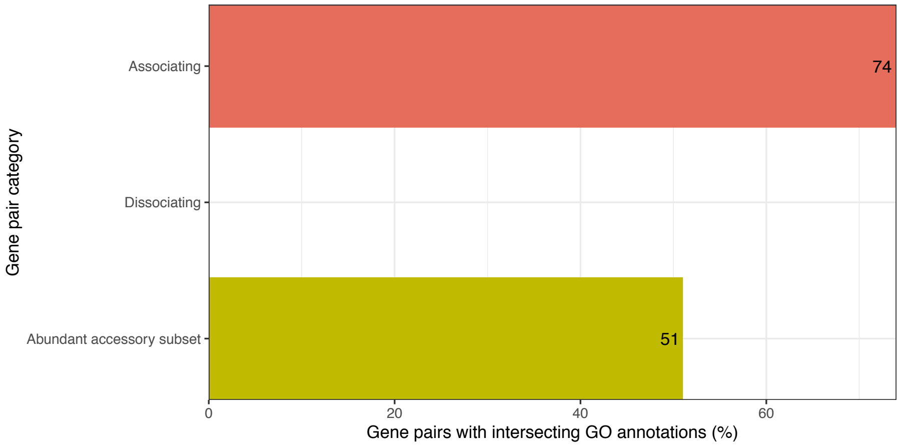

Supplementary Figure 8: **Non-syntenic coincident gene pairs share functionality.** Similar to the analysis of all coincident gene pairs (**Figure 3a**), the results hold when only those with a mean syntenic distance of ≥150,000bp between genes are considered. Because dissociating genes by definition are not found in the same genome, this analysis could not be performed on dissociating gene pairs.

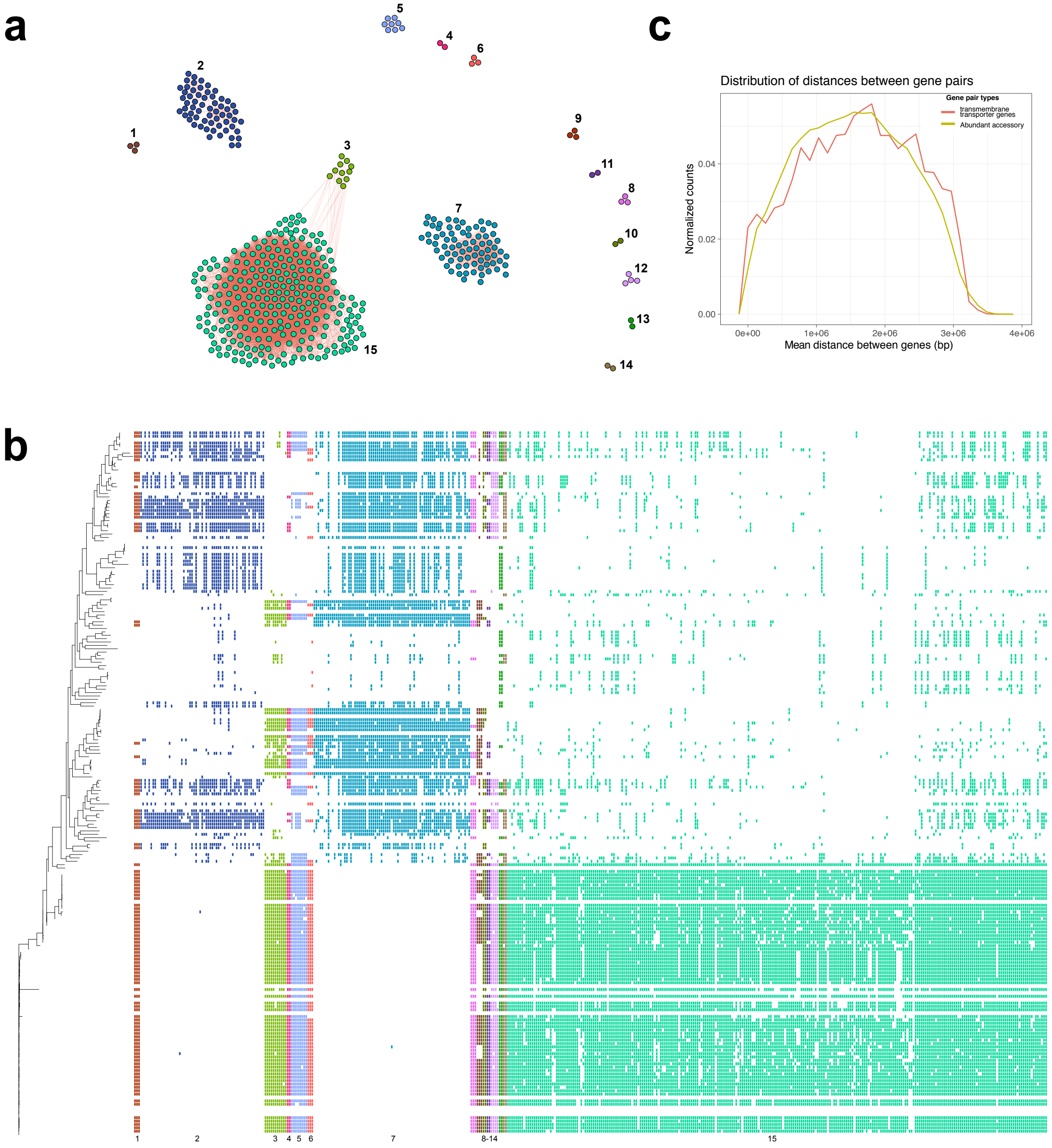

Supplementary Figure 9: **Genes annotated as transmembrane transporters are over represented in both gene association and dissociation relationships.** **a.** The gene association relationships amongst transmembrane transporters; this network is the same as that displayed in **Figure 3c** but with the dissociation edges hidden for visibility. Labels represent gene cluster numbers. **b.** The mean genomic distance between genes with the GO annotation of transmembrane transporters is evenly distributed across the pangenome. **c.** The presence absence patterns of the gene clusters across the *Pseudomonas sp.* phylogeny. Clusters are coloured and numbered as in part A and **Figure 3c**.

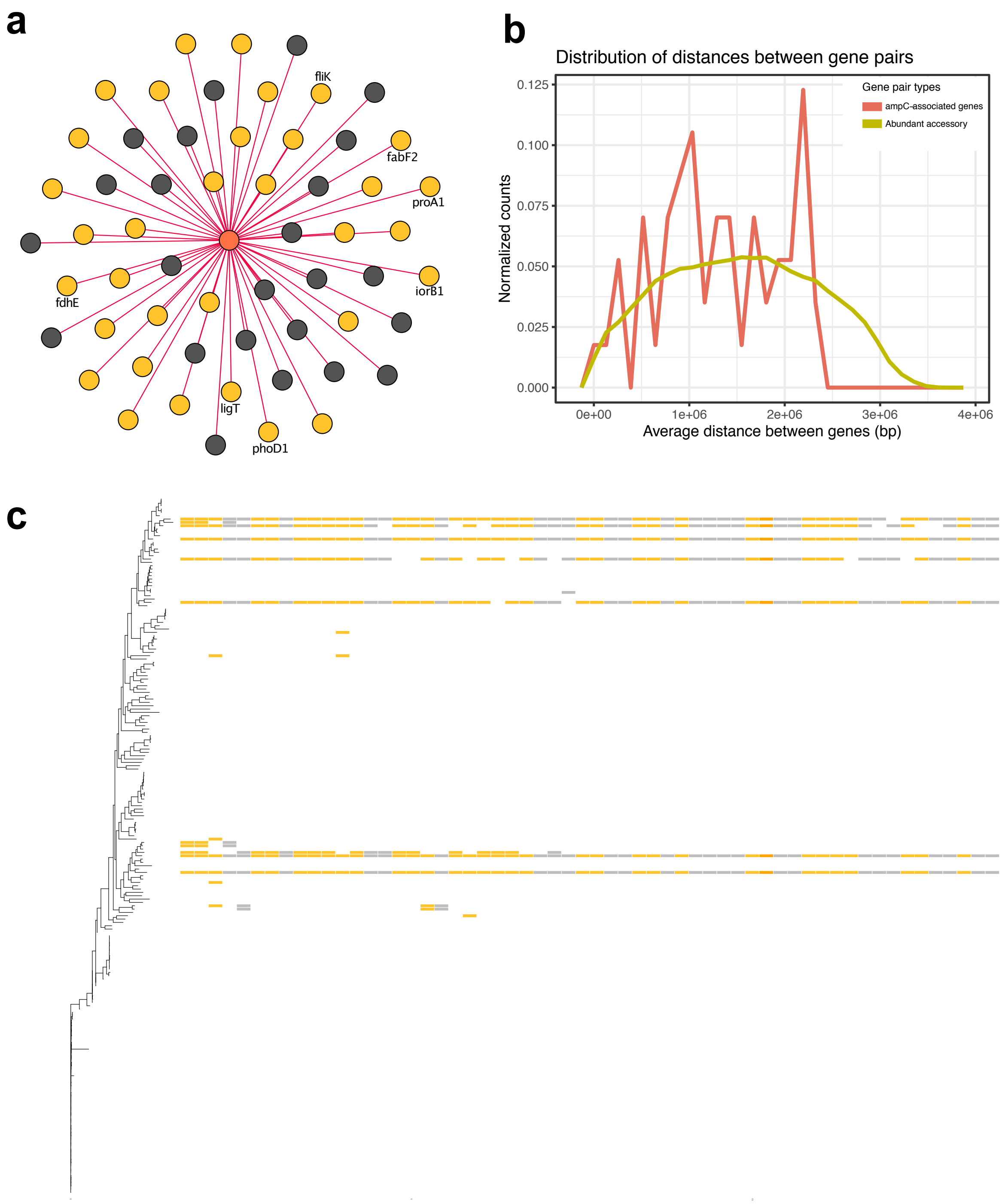

Supplementary Figure 10: **More hypothetical genes co-occur with an *ampC* homolog than would be expected by chance. a.** The network of genes associating with *ampC*. Genes with named annotations are labelled. **b.** These genes do not share significant co-localization with each other when compared with randomly chosen abundant accessory genes. **c.** The presence/absence patterns of these genes across the *Pseudomonas sp.* phylogeny indicates a lack of evolutionary history of these genes. Genes are coloured as to whether they are annotated (**yellow**) or hypothetical (**gray**). The *ampC* homolog is coloured **orange**.

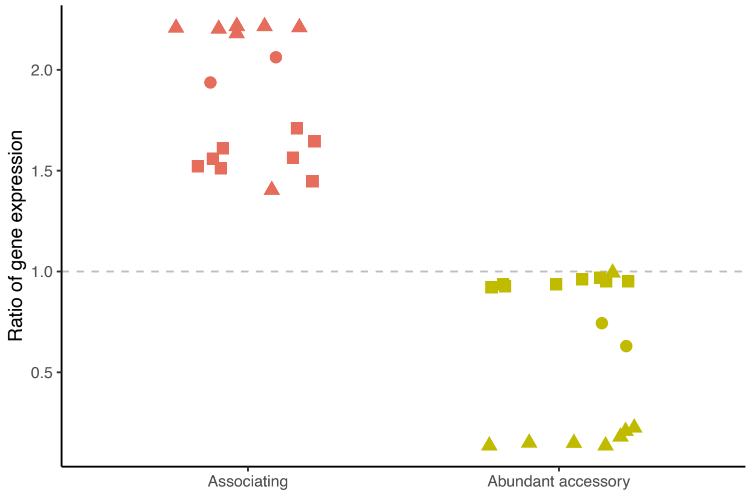

Supplementary Figure 11: **Gene co-expression ratios of non-syntenic gene pairs.** The gene co-expression ratio when only genes with $\geq$ 150,000bp between them were considered.

Supplementary Table 1: **Gene names and descriptions of the gene sets used as examples of high, low, and variable physical distance in Figure 2.** Gene cluster descriptions are based on the descriptions of each gene within the cluster; parentheses indicate how often a given description was observed.

Box #	Gene cluster ID	Gene name/description
1	group_31493	hypothetical protein (25); HAD family hydrolase (5); conserved hypothetical protein (1)
1	group_37342	nucleotidyltransferase (24); hypothetical protein (2); sugar nucleotidyltransferase (2); putative sugar nucleotidyltransferase (1)
1	group_49093	hypothetical protein (26); flagellar assembly protein FliT (3)
1	group_49094	flagellar protein FliS (23); flagellar biosynthesis protein FliS (3); B-type flagellar protein FliS (2); hypothetical protein (1)
1	group_49095	B-type flagellar hook-associated protein 2 (18); branched-chain alpha-keto acid dehydrogenase subunit E2 (7); flagellar capping protein FliD (4)
1	group_49096	hypothetical protein (29)
1	group_49097	glycosyl transferase family 1 (14); hypothetical protein (9); flagellar glycosyl transferase, FgtA (3); flagellar glycosyl transferase FgtA (2); glycosyl transferase (1)
1	group_49098	hypothetical protein (18); methyltransferase type 12 (10)
1	group_49099	flagellar hook-associated protein FlgL (28); flagellar hook-associated protein type 3 FlgL (1)
2	group_1102	hypothetical protein (40); dehydrogenase (17); aldose sugar dehydrogenase YliI (9); oxidoreductase (4); putative dehydrogenase (2); aldose sugar dehydrogenase (1); Soluble aldose sugar dehydrogenase, PQQ-dependent (1)
2	group_1963	porin (70); Glucose/carbohydrate outer membrane porin OprB precursor (6); Glucose-selective porin OprB (2); carbohydrate porin (1); outer membrane porin OprB (1)
2	group_7677	outer membrane protein OprG (66); outer membrane protein W (7); Outer membrane protein OprG precursor (5); Outer membrane protein W precursor (2); OmpW family protein (1)

3	group_11613	S-adenosylmethionine decarboxylase proenzyme (66); S-adenosylmethionine decarboxylase (13); S-adenosylmethionine decarboxylase proenzyme, prokaryotic class 1A (1)
3	group_6267	enolase-phosphatase E1 (30); haloacid dehalogenase (23); 2,3-diketo-5-methylthio-1-phosphopentane phosphatase (13); enolase-phosphatase E-1 (4); 2,3-diketo-5-methylthiopentyl-1-phosphate enolase-phosphatase (2); enolase-phosphatase (2)
3	group_7302	methylthioribulose-1-phosphate dehydratase (42); methylthioribulose 1-phosphate dehydratase (38); probable sugar aldolase (1)
3	group_7553	spermidine synthase (75); polyamine aminopropyltransferase 1 (6)

Supplementary Table 2: **Summary of statistically enriched GO terms in the association and dissociation datasets.** See SupTable2.xlsx.

Supplementary Table 3: **Summary of genes with transmembrane transporter activity over represented in both association and dissociation gene relationships.** See SupTable3.xlsx.

Supplementary Table 4: **Summary of statistically enriched GO terms with hypothetical pairs.** See SupTable4.xlsx.

Supplementary Table 5: **Summary of genes co-occurring with an *ampC* homolog.** Gene cluster descriptions are based on the descriptions of each gene within the cluster; parentheses indicate how often a given description was observed.

Gene cluster ID	Gene name/description
group_10590	hypothetical protein (13)
group_1110	spermidine/putrescine ABC transporter substrate-binding protein PotF (4); putrescine/spermidine ABC transporter substrate-binding protein (3); spermidine/putrescine ABC transporter substrate-binding protein (2); extracellular solute-binding protein (1); polyamine ABC transporter substrate-binding protein (1); putrescine-binding periplasmic protein (1)
group_13151	2'-5' RNA ligase (17)
group_13686	flagellar hook-length control protein (12); flagellar hook-length control protein FliK (2)
group_13726	hypothetical protein (16)
group_13727	twin-arginine translocation pathway signal protein (16); isoquinoline 1-oxidoreductase subunit beta (1)
group_13748	GNAT family acetyltransferase (10); GNAT family N-acetyltransferase (2); GCN5-like N-acetyltransferase (1); N-acetyltransferase GCN5 (1)
group_13811	hypothetical protein (14)
group_13818	phosphodiesterase (12); phosphodiesterase/alkaline phosphatase D-like protein (2)
group_13819	hypothetical protein (16)
group_13820	hybrid sensor histidine kinase/response regulator (8); histidine kinase (3); multi-sensor hybrid histidine kinase (2); hypothetical protein (1)
group_14115	formate dehydrogenase accessory protein FdhE (13); formate dehydrogenase accessory protein (1)
group_14332	glycosyltransferase (9); glycosyl transferase (3); hypothetical protein (1)
group_14389	aldo/keto reductase (14)
group_14455	peptide transporter (12); amino acid adenylation:thioester reductase (1)
group_14473	chorismate mutase (13)
group_15113	hypothetical protein (13)
group_15142	hypothetical protein (12)

group_15152	hypothetical protein (10); thiolase (2)
group_15235	DUF3077 domain-containing protein (9); hypothetical protein (5)
group_15241	serine protease (11); hypothetical protein (2)
group_15274	hypothetical protein (12)
group_15276	transcriptional regulator CynR (8); transcriptional regulator (4); DNA-binding transcriptional regulator CynR (1)
group_15277	hypothetical protein (14)
group_15280	ATPase (6); hypothetical protein (6)
group_15281	GCN5 family acetyltransferase (9); GNAT family N-acetyltransferase (2); GCN5-like N-acetyltransferase (1); N-acetyltransferase GCN5 (1)
group_15282	GDP-mannose pyrophosphatase (8); GDP-mannose pyrophosphatase nudK (2); nucleoside diphosphate pyrophosphatase (2); ADP-ribose pyrophosphatase (1)
group_15283	transcriptional regulator (11); Cro/CI family transcriptional regulator (1)
group_15284	hypothetical protein (10); delta-60 repeat domain-containing protein (2)
group_15285	hypothetical protein (11); lipoprotein (1)
group_15286	isochorismatase (10); cysteine hydrolase (1); isochorismatase hydrolase (1)
group_15287	calpastatin (9); hypothetical protein (3)
group_15887	polyketide cyclase (10); MxaD family protein (2); hypothetical protein (1)
group_15938	hypothetical protein (12)
group_16075	methylase (9); hypothetical protein (2); SAM-dependent methyltransferase (1)
group_16076	general secretion pathway protein GspH (10); general secretion pathway protein H (1)
group_16077	hypothetical protein (11)
group_16078	hypothetical protein (9); membrane protein (3)
group_16082	hypothetical protein (9); lipoprotein (2)
group_16083	hypothetical protein (12)
group_16084	hypothetical protein (11)
group_16085	hypothetical protein (12)
group_16086	hypothetical protein (12)
group_16087	NUDIX hydrolase (11)
group_16088	glycerol-3-phosphatase (10); HAD family hydrolase (1); phosphatase (1)

group_16091	acetyltransferase (9); GCN5-like N-acetyltransferase (1); GNAT family N-acetyltransferase (1)
group_16092	class C beta-lactamase (10); beta-lactamase (2)
group_16908	hypothetical protein (11)
group_17033	hypothetical protein (12)
group_17040	hypothetical protein (10); DUF4160 domain-containing protein (1)
group_17041	hypothetical protein (11); lipoprotein (1)
group_17048	glutamate-5-semialdehyde dehydrogenase (12)
group_17049	cupin (6); hypothetical protein (5)
group_17050	glutathione S-transferase (10); glutathione S-transferase-like protein (2)
group_18423	hypothetical protein (6); endoribonuclease L-PSP (4); endoribonuclease L-PSP family protein (1)
group_28806	(12); tRNA-Arg (3); tRNA-Arg;Dbxref=GeneID:14051617 (1); tRNA-Arg;Dbxref=GeneID:3712881 (1)
group_4768	beta-ketoacyl-[acyl-carrier-protein] synthase II (5); beta-ketoacyl-ACP synthase II (4); 3-oxoacyl-ACP synthase (2); 3-oxoacyl-(acyl carrier protein) synthase II (1)
group_6199	hypothetical protein (11)
